# Gluten‐free breadsticks with *Ganoderma* ‐fermented corncobs: technological and nutritional features

**DOI:** 10.1002/jsfa.14224

**Published:** 2025-03-18

**Authors:** Carola Cappa, Giulia Castorina, Giovanni Fiorillo, Maria Cristina Casiraghi, Manuela Rollini, Gabriella Consonni, Daniela Erba, Noemi Negrini, Alessandra Marti

**Affiliations:** ^1^ DeFENS, Department of Food, Environmental and Nutritional Sciences Università degli Studi di Milano Milan Italy; ^2^ DiSAA, Department of Agricultural and Environmental Sciences – Production, Landscape, Agroenergy Università degli Studi di Milano Milan Italy

**Keywords:** medicinal mushroom, solid‐state fermentation, phenols, dietary fiber, corncobs, starch digestibility

## Abstract

**Background:**

This study investigates the use of corncobs before and after fermentation with *Ganoderma annularis* (G) to enhance the nutritional value of gluten‐free breadsticks. Medicinal mushrooms are known to increase the nutrient profile of substrates through solid‐state fermentation (SSF); nevertheless, using the entire SSF – as adopted in this study – is unprecedented in gluten‐free baked goods. Corncobs from the B73 maize inbred line and the ‘Rostrato Rosso di Rovetta’ (RR) landrace were used.

**Results:**

During leavening, dough height increased by 1.5 times with RR and RR + G. Compared to the standard (STD) control sample, breadsticks containing 100 g kg^−1^ *Ganoderma*‐fermented corncobs exhibited a smaller diameter and increased breadstick breaking force (13.9 N for B73 + G and RR + G *versus* 7.6 N for STD). Corncob addition increased total fiber (88–100 *versus* 13 g kg^−1^ dry weight (DW) of STD) and reduced rapidly digested starch (11% lower than STD) of breadsticks; fermented corncobs also increased soluble dietary fiber (5 *versus* 1 g kg^−1^ DW). The addition of unfermented or fermented corncobs to breadsticks enhanced total phenol content (from 0.2 to around 3 mg gallic acid equivalent (GAE) g^−1^ DW) and antioxidant capacity (from 0.3 to 8 μmol ascorbic acid equivalent g^−1^ DW). RR and RR + G breadsticks showed the highest content of free phenols (0.40 and 0.32 mg GAE g^−1^ DW, respectively).

**Conclusion:**

The addition of *Ganoderma*‐fermented corncobs to gluten‐free breadsticks increases fiber and antioxidant content, offering potential health benefits. The contribution of bioactive ingredients with beneficial effects, made by the RR landrace, deserves further investigation. © 2025 The Author(s). *Journal of the Science of Food and Agriculture* published by John Wiley & Sons Ltd on behalf of Society of Chemical Industry.

## INTRODUCTION

Maize (*Zea mays*) is the most widely produced cereal, with production of about 1.16 million tons in 2022, followed by wheat and rice, and is the second most widely produced commodity in the world after sugar cane.^1^ 61% of the global maize production is used as animal feed and 13% is valued for food production; the remaining 26% is used for other purposes, including biofuel production.^2^, ^3^ The maize kernel, with a high content of starch, protein and oil, as well as some vitamins and minerals, is the edible part of the plant and is the main product harvested from this crop. However, a wide range of agricultural residues are produced during grain harvesting, including husks (about 8%), leaves (about 19%), stalks (about 58%) and corncobs (about 12%).^4^ Specifically, the average yield of corncobs, the central cylindrical parts of the maize ear to which the kernels are attached, is estimated to be around 14% of grain yield, with the latter reaching 4–6 tons ha^−1^ in 2022.^1^ Corncobs are generally discarded or left unused, causing a waste of resources and an environmental issue.^5^ As corncobs are mainly composed of hemicellulose (33–43%), cellulose (26–36%) and lignin (17–21%),^6^ their main application is as animal feed and, above all, as a substrate for the production of bioenergy, enzymes (i.e. cellulases, xylanases, pectinases) and other components, such as xylooligosaccharides, lactic acid and pigments.^7^, ^8^

More recently, corncobs have been used as a substrate for the growth of edible medicinal mushrooms (MMs) by solid‐state fermentation (SSF).^9^, ^10^ SSF is a biotechnological process that involves the use of solid materials, generally food byproducts or food losses, where mycelial growth occurs in the presence of low amounts of water (50–75%).^11^ The combination of this technique with the growth of MMs on agro‐industrial residues, such as corncob, represents a valid alternative to their disposal, which would have a negative environmental impact. Moreover, the use of MMs as inoculum is an effective approach for enhancing the nutritional value of the substrate, mushrooms being a valuable source of fiber, proteins, essential amino acids, polyunsaturated fatty acids (i.e. linoleic and oleic acids), vitamins (i.e. B_1_, B_2_, B_3_), minerals (i.e. iron and calcium) and phenolic compounds.^12^, ^13^

Several studies have investigated the potentiality of producing novel culinary MM carpophores mainly by the genus *Pleurotus* grown on SSF cultures to produce food products with improved nutritional value; nevertheless, to the best of our knowledge none of them proposed the use of the entire solid culture (i.e. the substrate fermented with the MMs) in the food sector.^14^, ^15^, ^16^, ^17^

In a previous study,^10^ we investigated the use of corncobs from different corn genotypes (i.e. the reference inbred line B73, and four distinct maize landraces, namely ‘Fiorine di Clusone’, ‘Spinato di Gandino’, ‘Rostrato Rosso di Rovetta’ and ‘Spinoso Nero Valle Camonica’) as substrates for implementing SSF processes with various MMs (i.e. *Pleurotus ostreatus*, *Ganoderma annularis*, *Flammulina velutipes* and *Lentinula edodes*). Overall, the growing capacity and the chemical composition of the biomass depend on both the genotype and the fungal strains, with *Ganoderma annularis* showing the most promising results in terms of growth rate and nutritional features of the resulting biomass, in terms of increased proteins (up to 60 g kg^−1^) and soluble fiber (up to 50 g kg^−1^). Although the main outcome of the study was the production and characterization of a new ingredient, no information on the impact of using fermented corncobs on product quality is available.^10^ Thus, the study reported here aimed at evaluating the effect of the addition of *Ganoderma*‐fermented corncobs on the technological and nutritional properties of gluten‐free baked product.

## MATERIALS AND METHODS

### Chemicals

All chemicals were purchased from Sigma Aldrich (Milan, Italy). The culture medium potato dextrose agar (PDA) was purchased from Formedium (Swaffham, UK). K_2_HPO_4_, (NH_4_)_2_SO_4_ and MgSO_4_ were from VWR International srl (Milan, Italy). Yeast extract was purchased from Costantino SpA (Turin, Italy) and glucose from Duchefa (Haarlem, Netherlands).

### Microbial strain

The strain *Ganoderma annularis* DSMZ 9943 (Deutsche Sammlung von Mikroorganismen und Zellkulturen GmbH, Braunschweig, Germany) used in this study was maintained on 5 cm plates containing PDA culture medium. Inoculum was performed by depositing a quarter (around 5 cm^2^) of an older (maximum 2 months) solid culture plate to the surface, using a sterile scalpel. The plates were subsequently incubated at 25 °C in the dark. When the mycelium completely covered the surface of the solid culture, the plates were tightly sealed with Parafilm and stored at 4 °C for a maximum of 2 months before use.

### Plant material

The corncobs were obtained from plants of two maize (*Zea mays* L. ssp. *mays*) genotypes: the inbred line B73 and the landrace ‘Rostrato Rosso di Rovetta – VA1306’ (RR). The seeds of the B73 line and the RR landrace were originally provided by the Maize Genetics Cooperation Stock Center (http://maizecoop.cropsci.uiuc.edu) and the CREA Bergamo germplasm bank (www.ecpgr.cgiar.org/working-groups/maize/maize-wg), respectively. Maize plants of both genotypes were grown during the summer 2022 in an experimental field at Università degli Studi di Milano, in accordance with organic agronomic practices, and were reproduced via sibling mating. The ears were manually harvested at maturity, shelled and dried to achieve a moisture content of 120–130 g kg^−1^. Following the removal of the seeds, the corncobs were stored at room temperature until research analyses.

The authors confirm that the use of plants in the present study complies with international, national and/or institutional guidelines.

### Corncob fermentations in solid state (SSFs)

Boxes (16 × 10 cm^2^) equipped with a filtering air system (Berry Superfos, Taastrup, Denmark) were filled with 50 g of ground corncobs (B73 or RR), chopped to a maximum size of 0.5 cm first with a knife then with a Bimby 300 (Worwerk, Wupperthal, Germany) at speed 7 for 1 min. Boxes were then sterilized at 117 °C for 20 min and then added with 100 mL of a mineral solution containing (g L^−1^): K_2_HPO_4_, 1; (NH_4_)_2_SO_4_, 5; MgSO_4_, 0.2; yeast extract, 1; adjusted to a pH of 5.8. Two batches of SSF cultures were prepared to obtain the material required for breadstick production. As described by Castorina *et al*.,^10^ the solid cultures were inoculated with 20 mL of a liquid *Ganoderma* pre‐culture, then incubated at 25 °C in the dark for 42 days, monitoring mycelia growth by an image analysis technique. *Ganoderma*‐fermented B73 and RR corncobs were named B73 + G and RR + G, respectively.

### Culture stabilization and milling

After incubation, *Ganoderma*‐fermented corncobs were dried at low temperature (50 ± 2 °C) in a vacuum oven (WIPA, GEASS, Turin, Italy; operating at 0.987 Pa) to reach a moisture content in the range of 150–180 g kg^−1^ and ground at room temperature using a disc mill (MLI 204, Buhler, Italy) to pass through a screen with 1.0 mm openings. The resulting powders were packed in plastic bags and stored in the dark at 4 °C until being used for breadstick production.

### Gluten‐free breadstick preparation and grinding

Breadstick samples (at least 25 for each formulation) were prepared following an internal procedure developed to produce gluten‐free samples shapeable at the laboratory level. Control breadsticks (STD) were prepared using rice flour (RF; Molino F.lli Chiavazza SpA, Italy), while for the other samples 100 g kg^−1^ of RF was singularly substituted with unfermented (B73 or RR) or *Ganoderma*‐fermented corncobs (B73 + G or RR + G). The amount of the other ingredients was kept constant among all samples, as follows: 150 g kg^−1^ extra virgin olive oil (Farchioni Olii SpA, Italy), 30 g kg^−1^ dry yeast (Carrefour Italia SpA, Italy), 30 g kg^−1^ salt (Carrefour Italia SpA, Italy), 10 g kg^−1^ hydroxypropylmethylcellulose (UNIVAR SpA, Italy). Distilled water was added to reach a target consistency (75 ± 5 Brabender Unit, BU) that allowed the dough to be poured in molds avoiding sample cracking during baking. For each formulation, breadsticks were prepared by firstly suspending salt and yeast in two separate aliquots of warm water (35 ± 2 °C). Then, these dissolved ingredients were added to the pre‐mixed dry ingredients and kneaded using a mixer (Kenwood KM020, Kenwood Manufacturing Co., UK) equipped with a flat beater. To allow for complete hydration of the powdered ingredients, olive oil was added as the last ingredient. Kneading was conducted at speed 4 for 10 min.

After mixing, the dough was proofed for 60 min. The first 30 min of leavening was carried out at 35 °C in a leavening oven (Whirlpool AMW 698/IXL, Whirlpool EMEA SpA, Italy) at 80% relative humidity; then, the partially leavened dough was manually portioned (6.5 ± 0.5 g for each breadstick) using a sac‐à‐poche equipped with a ‘V’ opening into a breadstick silicone mold (Lekue, UK) previously treated with release spray (Rebecchi F.lli Valtrebbia SpA, Italy). Shaping was carried out at room temperature (25 °C) for 30 min and the dough leavening was allowed to complete. Lastly, gluten‐free breadsticks were baked in a pre‐heated electric oven (AKPM 759/XL, Ignis Whirlpool Srl, Italy) in fan mode, following a two‐step baking process: they were firstly baked at 200 °C for 15 min, allowed to cool at room temperature for 2 min, turned upside down and baked again for other 15 min at 200 °C. At an industrial level, a one‐stage cooking process is often used; however, based on preliminary tests conducted at the laboratory scale, when a one‐stage cooking was used the breadsticks exhibited significant cracking and were not cooked evenly, with the bottoms remaining undercooked and the tops overcooked. Therefore, according to Conte *et al*.,^18^ a two‐stage cooking was preferred.

After baking, breadstick samples were cooled for 15 min into a hermetic container, before analysis. Breadsticks were ground at low speed for 10 s in a blender (Braun Multiquick System ZK100, Germany). Then the powdered samples were directly analyzed or stored at −20 °C until further characterization.

### Proximate composition

The proximate composition was analyzed on ground breadsticks, following established official methods for moisture (AACC 44‐15.02), crude protein (AACC 46‐12.01), crude fat (AACC 30‐10.01) and ash (AACC 08‐01.01) content.^19^ Total starch content was determined by using AACC method 76‐13.01 (K‐TSTA kit, Megazyme, Wicklow, Ireland)^19^ and free sugars by HPLC, according to the method of Rocklin and Pohl.^20^ Total (TDF), soluble (SDF) and insoluble (IDF) dietary fiber fractions were analyzed by the official method (AACC 32‐07.01).^19^ Resistant starch (RS) was analyzed by AACC method 32‐40.01 (K‐RSTAR kit, Megazyme, Wicklow, Ireland).^19^ Analyses were performed in triplicate and results expressed as mean ± standard deviation (SD).

### Technological properties of dough and breadsticks

#### Dough properties

The mixing properties of doughs were investigated with a Brabender Farinograph (Brabender® OHG, Duisburg, Germany) set at 30 °C. The ingredients were mixed directly in the instrument chamber (50 g bowl) in the order previously reported and kneaded for 10 min. Water absorption (g kg^−1^; amount of water required to reach the desired consistency after 10 min of mixing; i.e. 75 ± 5 BU) was determined. Dough pH was measured with a pH meter (S20, SevenEasy, Mettler Toledo, Switzerland) on 10 g of dough at 25 °C. To evaluate the leavening behavior, the rise of about 20 g of dough over time was evaluated in 100 mL graduated cylinders at 35 °C every 10 min up to 60 min, then dough volume increase (%) was calculated at each resting time. For each formulation, dough was produced twice and two subsamples from each dough were analyzed (*n* = 4).

#### Breadstick properties

Baking loss (g kg^−1^) was computed as the difference between the weight of the dough before baking and the weight of the cooled breadsticks with respect to the dough weight. Results are the average of at least 25 measurements.

Geometrical characteristics (i.e. breadstick and breadstick section dimensions) were investigated using image analysis. For each formulation, the image of 10 breadsticks as is and sectioned in two parts were acquired at 600 dpi resolution and 24‐bit color depth using an Epson Perfection V850pro scanner (Seiko Epson Corporation, Suwa, Japan). Images were processed using specific software (Image Pro‐Plus 7.0; Media Cybernetics Inc., Rockville, MD, USA) and the objects (i.e. breadsticks) were identified and measured in terms of breadstick length (mm) and major (mm) and minor (mm) axes of the sample section. Results are an average of 10 measurements.

The colorimetric indices were determined on ground samples (approximately 30 g) levelled in Petri dishes using a Minolta Chroma Meter II (Minolta, Osaka, Japan) equipped with standard Illuminant C and previously calibrated using the standard white reflector plate (*Y* = 87.7; *x* = 0.308; *y* = 0.315). Results were expressed in the CIE L* a* b* space: L* (lightness; from black (0) to white (100)), a* (from green (−) to red (+)) and b* (from blue (−) to yellow (+)). Δ*E* values with respect to STD sample were calculated using the following equation:

$$\Delta E=\sqrt{\left({\Delta L}^{2}+{\Delta a}^{2}+{\Delta b}^{2}\right)}$$

Six measurements (*n* = 6) were carried out for each breadstick recipe.

Breadstick moisture (g kg^−1^) was determined gravimetrically on powdered breadsticks after incubation at 105 °C until constant weight, while water activity (*a*_w_) was determined at 25 °C using an Aqualab Series CX‐3 device (Decagon Devices Inc., USA) previously calibrated with a standard solution (*a*_w_ = 0.250). Results are the average of three measurements.

Breadstick textural properties were determined as reported by Alamprese *et al*.^21^ Briefly, a three‐point bending test was carried out at room temperature using a TA.HDplus texture analyzer (Stable Micro Systems Ltd, UK) equipped with a 500 N load cell and controlled through specific software (Texture Exponent TEE32, v. 3.0.4.0, Stable Micro Systems Ltd, UK). One breadstick (10 cm long) per time was placed centrally over the appropriate device (HDP/3PBeThree Point Bend) with a 61 mm span length and broken by a blade probe moving downwards at a pre‐test speed of 5 mm s^−1^ and a test speed of 10 mm s^−1^. The maximum peak force (N) required to break the sample and the distance to break (mm) were determined from the obtained force–distance curves and further referred to as hardness and brittleness. Results are an average of 20 measurements.

### *In vitro* starch digestibility

Rapidly (RDS) and slowly (SDS) digestible starch fractions were determined using the method of Englyst *et al*.^22^ Breadstick samples were minced to simulate mastication, thus they were passed in a mincer, manually cranked with a plate of 0.9 cm diameter holes.^23^ Aliquots containing 500–600 mg of starch were digested simulating gastric and intestinal phases of the human digestion process (first with pepsin P7000, and after with a mixture of pancreatin P7545 and amyloglucosidase A7095; Sigma Chemical Co., St Louis, MO, USA) under controlled conditions. Aliquots were taken from the incubation mixture at 20 and 120 min after the start of the hydrolysis, and their glucose concentrations were analyzed by HPLC.^20^ The amount of glucose released at 20 min was used to calculate the RDS and that at 120 min the SDS. RDS and SDS were expressed as percentage of available starch (RDS + SDS)^22^ and as contents of RDS and SDS per kilogram dry weight (DW) of breadsticks. Analyses were performed in triplicate.

### Free and bound phenolic determination

Free and bound phenolics from ground breadsticks were extracted in triplicate according to Castorina *et al*.^10^ Total phenolic content in both free and bound phenolic fractions was assayed according to the Folin–Ciocalteu method with some modifications.^10^ Results were expressed as milligrams of gallic acid equivalents (GAE) per gram DW.

### Antioxidant activity against DPPH

Antioxidant capacity of the free and bound phenolic fractions was determined using the free radical 2,2‐diphenyl‐1‐picrylhydrazyl (DPPH) inhibition antioxidant assay, according to the literature.^24^, ^25^ The assay was performed as previously reported^10^ and the total antioxidant capacity was expressed as μmol of ascorbic acid equivalents (ASAE) per gram DW.

### Statistical analyses

Analysis of variance (ANOVA) was carried out using Statgraphics Plus 5.1 (StatPoint Inc., Warrenton, VA, USA) to determine significant (*P* < 0.05) differences among the samples. When a factor effect was found to be significant, significant differences among the respective averages were determined using Tukey's honest significant difference test.

## RESULTS AND DISCUSSION

The conditions adopted for the cultivation of *Ganoderma* were effective for the purpose of the study, which was the production of a substrate/biomass matrix to be used as an ingredient in baked goods. The impossibility of separating the mycelium from the substrate made the estimation of biomass growth challenging. Fungal mycelium deeply penetrated into corncobs, becoming inextricably entangled within the solids, merging into a single matrix. As reviewed by Manan and Webb,^26^ in SSF trials indirect methods for estimating mycelial growth can be applied. No method is ideally suited to all situations, but the most appropriate must be chosen in each case based on the simplicity of the procedure, its cost and accuracy. The use of image analysis in the present research had the benefit of being practical, reliable and not too time‐consuming. Once standardized image acquisition, this procedure allowed the comparison of fungal growth rate at the surface of SSF in the presence of corncobs coming from different local landraces.^10^ Even if the growth under the surface is not monitored, growth of aerobic organisms occurs at its best at the surface where oxygen is available.^27^ In the present trials, *Ganoderma* covered 100% of the surface of the SSFs prepared with either B73 or RR corncobs after 42 days of incubations, in line with previously reported results.^10^

**Figure 1:**
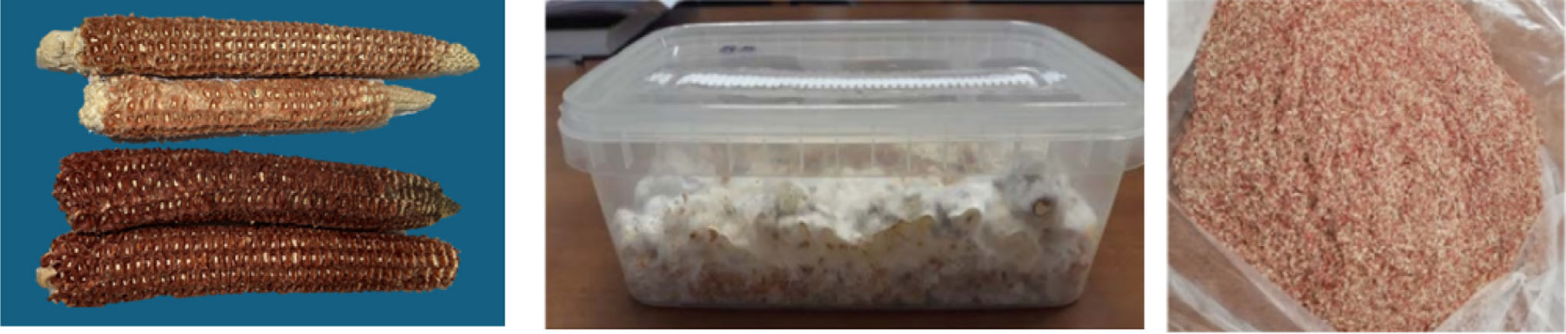
From left to right: images of the two corncob types (B73, top; RR, bottom); *Ganoderma annularis* (G)‐fermented corncobs; new *Ganoderma*–RR‐fermented ingredient after milling.

Fermented culture samples were then dried at low temperature (50 ± 2 °C under vacuum) to achieve a final moisture content of 150–180 g kg^−1^ and ground to obtain a functional ingredient (Fig. 1) to use in gluten‐free breadstick production.

### Technological properties of dough and breadstick samples

Breadsticks were prepared by incorporating 100 g kg^−1^ of both unfermented and fermented corncobs into rice flour. Given the lack of studies on the use of *Ganoderma*‐fermented corncobs (or other byproducts) in baked goods, we referred to research conducted on biscuits which demonstrated that mushroom substitution levels of up to 100 g kg^−1^ were effective in producing items with desirable nutritional qualities.^28^, ^29^

To compare the performance of dough prepared with different ingredients, two strategies can be applied: (1) to add the same amount of water to all the ingredients regardless of their water affinity; (2) to prepare dough having the same consistency (i.e. farinographic consistency) by adding the water amount required by each ingredient. This second strategy was chosen to balance out moisture and water affinity differences among ingredients. Liquid‐like dough (i.e. similar to a batter) is commonly used for gluten‐free production instead of the 500 BU of gluten‐containing dough^30^, ^31^; thus, after some laboratory trials focused on guaranteeing a proper shaping of the dough, a farinographic dough consistency of 75 ± 5 BU was chosen. As expected, to reach the desired dough consistency different amounts of water were necessary (g kg^−1^): 440 for STD, 472 for B73 and RR, 442 for B73 + G and RR + G; this can be partially attributed to the moisture content of the unfermented or fermented corncob powders (approximately 65 and 180 g kg^−1^, respectively) and their composition (e.g. protein and fiber content). No significant (*P* > 0.05; statistical analysis not shown) differences among dough consistencies (73–80 BU) were detected and doughs showed a similar theoretical water content (500 ± 10 g kg^−1^); pH was also slightly affected by corncob addition, that for both fermented and unfermented ranging between 5.41 and 5.52 *versus* 5.59 of STD dough.

**Figure 2:**
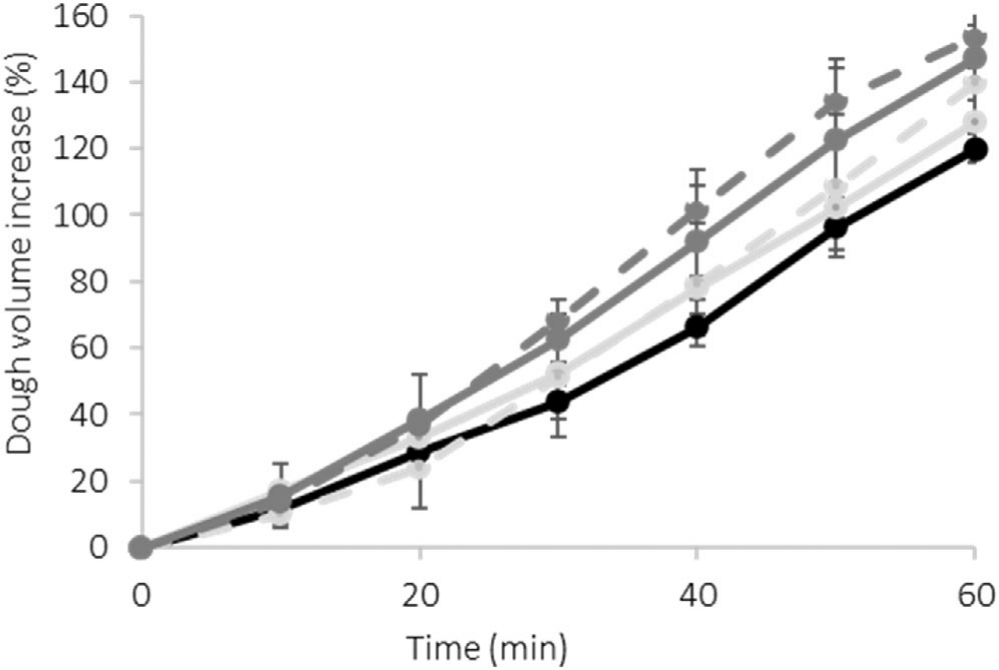
Dough leavening properties over time, expressed as volume increase. Black solid line, STD sample not containing corncobs; light grey solid line, sample with B73 corncobs; light grey dotted line, with *Ganoderma*‐fermented B73 corncobs; grey solid line, sample with RR corncobs; grey dotted line, with *Ganoderma‐*fermented RR corncobs (data are reported as mean ± SD, *n* ≥ 4).

The height of STD dough (i.e. the sample without corncobs) increased by 120% during 60 min of leavening (Fig. 2). Adding B73 corncobs into the formulation (either before or after fermentation with *Ganoderma*) did not significantly (*P* > 0.05; statistical analysis not shown) affect dough development (that increased after 60 min by 128% and 140% for B73 and B73 + G, respectively). On the other hand, when RR corncobs were used, a significant (*P* < 0.05; statistical analysis not shown) increase in dough height was measured and specifically, at the end of leavening, dough height increased up to 147% and 153% for RR and RR + G, respectively.

The impact of potential differences in dough consistency or theoretical dough moisture were excluded, due to similarity among samples as already discussed above. Moreover, the differences in chemical composition (e.g. sugar and fiber content) observed in corncob ingredients^10^ can partially explain dough behavior, suggesting that also aspects other than composition (e.g. starch‐fiber/lignin/phenolic compound interactions) should be considered to explain the differences in dough development during leavening. Although the fermentation of corncobs did not result in a significant impact on dough development, dough samples enriched in *Ganoderma*‐fermented corncobs exhibited a slightly greater height increase compared to the unfermented corncob‐enriched doughs. This behavior may be in part due to the presence of hydrolytic enzymatic activities, such as cellulases, amylases and amyloglucosidase in *Ganoderma*‐fermented cobs, which were not completely inactivated by the mild drying treatment applied after fungal growth. The conditions applied during the leavening step might have reactivated the enzymatic activity present in fermented corncobs. Specifically, it is recognized that amylases quickly hydrolyze the damaged starch and xylanases act on the fiber present in wheat flour. The presence of these enzymatic activities results in an increase of low‐molecular‐weight saccharides fermentable by *S. cerevisiae* and consequently, the dough height increases compared to the respective non‐*Ganoderma*‐fermented samples. As reported by Santiago *et al*.,^32^ various enzymes for bread making are used, especially *α*‐amylase and xylanases, to increase fermentable sugars, the formation of a desirable gluten network, an increased specific loaf volume and a retarded bread staling rate during storage.

**Figure 3:**
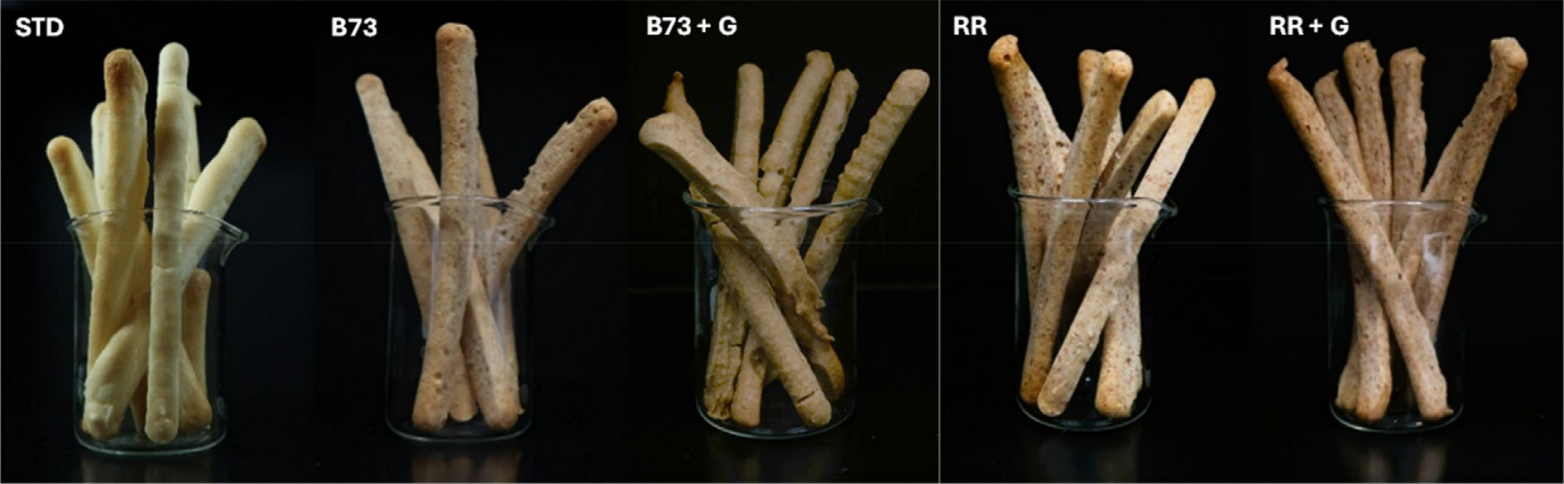
Images of breadstick samples. STD, breadsticks not containing corncobs; B73, breadsticks with B73 corncobs; B73 + G, breadsticks added with *Ganoderma‐*fermented B73 corncobs; RR, breadsticks with RR corncobs; RR + G, breadsticks added with the *Ganoderma*‐fermented RR corncobs.

Images of breadsticks are reported in Fig. 3, while the geometric, colorimetric and physical indices are summarized in Table 1. The addition of corncobs slightly affected the geometric indices of gluten‐free breadsticks, except for sample B73 + G, whose length was significantly (*P* < 0.05) lower than that of STD. Furthermore, compared with STD, samples prepared with the *Ganoderma*‐fermented corncobs (regardless of corncob type) exhibited a smaller diameter (measured as major and minor axis of the cross‐section), which did not differ from that of samples prepared with unfermented corncobs. These observations suggest that the substitution of 100 g kg^−1^ of RF with corncobs modified the rheological properties of dough (e.g. viscous properties) and consequently the breadstick diameter; this can be explained by a different total dietary fiber content as reported in the following (Table 2) and the ingredient affinity to water.

**Table 1:** Geometrical attributes, color indices, water activity and texture of breadsticks samples

Parameter	STD	B73	B73 + G	RR	RR + G
Length (mm)	102.3 ± 0.8^b^	101.6 ± 1.3^ab^	99.4 ± 4.0^a^	101.3 ± 1.0^ab^	101.2 ± 1.2^ab^
Section: major axis (mm)	12.8 ± 0.6^b^	11.7 ± 0.5^b^	10.3 ± 0.9^a^	11.6 ± 1.2^b^	10.1 ± 1.4^a^
Section: minor axis (mm)	9.0 ± 0.7^b^	8.3 ± 0.4^ab^	7.7 ± 1.0^a^	8.3 ± 0.4^ab^	7.7 ± 0.5^a^
L*	79.9 ± 0.5^d^	72.9 ± 0.7^c^	63.1 ± 1.1^a^	64.4 ± 1.3^b^	62.6 ± 0.9^a^
a*	−1.5 ± 0.2^a^	1.1 ± 0.4^b^	3.2 ± 0.5^c^	4.0 ± 0.4^d^	2.7 ± 0.4^c^
b*	22.3 ± 0.6^b^	23.4 ± 0.4^c^	24.4 ± 0.5^d^	20.6 ± 1.0^a^	21.3 ± 0.6^a^
Moisture (g kg^−1^)	81.8 ± 0.6^c^	77.1 ± 0.2^b^	77.2 ± 0.2^b^	90.9 ± 3.5^d^	63.9 ± 0.2^a^
Water activity	0.520 ± 0.027^c^	0.476 ± 0.013^b^	0.508 ± 0.007^bc^	0.566 ± 0.023^d^	0.373 ± 0.009^a^
Breaking force (N)	7.6 ± 1.5^a^	10.9 ± 1.5^b^	13.9 ± 2.3^c^	9.2 ± 1.2^b^	13.9 ± 3.0^c^
Fracture strength (mPa)	0.96 ± 0.20^a^	1.82 ± 0.20^b^	2.75 ± 0.40^c^	1.75 ± 0.27^b^	3.55 ± 0.68^d^
Breaking distance (mm)	0.65 ± 0.18^ab^	0.67 ± 0.17^ab^	0.85 ± 0.18^c^	0.72 ± 0.15^b^	0.60 ± 0.17^a^

Within the same row, data having different letters are significantly different (*P* < 0.05). Data are reported as mean ± SD (*n* ≥ 3).

B73 + G, breadsticks added with *Ganoderma*‐fermented B73 corncobs; B73, breadsticks with B73 corncobs; RR + G, breadsticks added with the *Ganoderma*‐fermented RR corncobs; RR, breadsticks with RR corncobs; STD, breadsticks not containing corncobs.

**Table 2:** Proximate composition (g kg^−1^ DW) of breadstick samples

	STD	B73	B73 + G	RR	RR + G
Crude protein	78 ± 1.2^a^	76 ± 1.5^a^	79 ± 1.0^a^	78 ± 2.1^a^	79 ± 4.4^a^
Lipid	145 ± 0.3^c^	137 ± 6.1^bc^	123 ± 0.8^a^	129 ± 1.1^ab^	144 ± 3.8^c^
Free sugar	2.0 ± 0.1^a^	2.0 ± 0.2^a^	2.0 ± 0.1^a^	2.0 ± 0.1^a^	2.0 ± 0.2^a^
Total starch	737 ± 21.9^b^	639 ± 24.8^a^	648 ± 15^a^	663 ± 8.8^a^	657 ± 14.7^a^
Ash	31 ± 0.3^a^	31 ± 0.1^a^	33 ± 0.9^b^	31 ± 0.3^a^	32 ± 0.5^ab^
Total dietary fiber (TDF)	13 ± 2.4^a^	95 ± 5.0^bc^	100 ± 3.9^c^	91 ± 0.4^b^	88 ± 1.8^b^
Insoluble dietary fiber (IDF)	10 ± 3.2^a^	92 ± 4.3^c^	93 ± 2.9^c^	87 ± 0.6^bc^	80 ± 1.3^b^
Soluble dietary fiber (SDF)	0.6 ± 1.1^a^	0.4 ± 0.8^a^	5.0 ± 0.9^b^	0.7 ± 0.5^a^	6.2 ± 1.9^b^
Resistant starch	2.2 ± 0.6^a^	2.5 ± 0.6^a^	2.1 ± 0.1^a^	2.4 ± 0.8^a^	2.2 ± 0.3^a^

In the same row, data not sharing common letters are significantly different (*P* < 0.05).

B73 + G, breadsticks added with *Ganoderma*‐fermented B73 corncobs; B73, breadsticks with B73 corncobs; RR + G, breadsticks added with *Ganoderma*‐fermented RR corncobs; RR, breadsticks with RR corncobs; STD, breadsticks not containing corncobs.

As regards the product color, luminosity decreased in the presence of corncobs and especially when *Ganoderma*‐fermented corncobs were used. The decrease in L* indicates that samples became darker with the addition of corncobs, likely due to their higher fiber content (Table 2) and the intrinsic color of the corncob powder added (see Fig. 1 as an example). For the same reason, an overall increase in the degree of redness was observed for the new products. However, the changes in a* were influenced by the substrate: in fact, the redness value was higher in breadsticks containing B73‐fermented corncob but lower in those containing RR‐fermented corncob than in products prepared with the corresponding unfermented corncobs. The increase in a* values in bakery products is generally associated with the Maillard reaction, as discussed in detail by Giovanelli and Cappa^33^; however, in the current study the baking condition was kept constant for all breadstick production and the raw material composition data (i.e. sugar and protein) reported by Castorina *et al*.^10^ do not seem to support the hypothesis of a more intense Maillard reaction when fermented corncobs are used as ingredients in the formulation of breadsticks. The degree of yellowness followed the order RR = RR + G < STD < B73 < B73 + G, indicating that this parameter was significantly affected by both the type of corncob and the fermentation process. However, Δ*E* values calculated for new breadsticks *versus* STD sample ranged between 17.6 and 24.9 confirming that unique colors can be obtained with 100 g kg^−1^ addition of corncob powder, both fermented and not fermented, and color changes were visible to the human eye as Δ*E* values were above 3.^34^

Finally, moisture content and water activity values (Table 1) were higher than values reported by Rainero *et al*.^34^ for gluten‐containing breadsticks added with red grape pomace (26–30 g kg^−1^ and 0.2, respectively, for moisture and *a*_w_) but consistent with this type of bakery product, and they can guarantee – from a microbiological point of view – a long shelf‐life at ambient conditions.

As regards breadstick texture, even if generally the increase in fiber content in gluten‐free product resulted in a weakness of the protein network,^35^, ^36^ addition of corncobs (and especially fermented corncobs) was responsible for the formation of a more compact structure that required a higher force to break the breadsticks, even normalizing force by sample dimension (i.e. fracture strength); these differences can be attributed to the diversity of the products: indeed, bread has a soft alveolar crumb, whereas breadstick has a harder alveolar internal structure. Alamprese *et al*.^21^ reported lower values for the fracture strength (3.5 ± 0.4 mPa) for whole‐wheat breadsticks. Differences in formulation (i.e. wheat‐based *versus* gluten‐free product) might be responsible for differences in results.

Another important texture parameter is the distance crossed by the blade through the sample before its breaking (i.e. breaking distance). According to previous studies^21^, ^37^, ^38^ brittleness behavior is preferred for breadsticks. Excluding B73 + G, addition of corncobs resulted in breaking distance values not significantly (*P* > 0.05) different from those for STD, suggesting that corncob powder can be added without extensively penalizing breadstick structure.

### Proximate composition of gluten‐free breadsticks

Proximate composition of breadsticks is reported in Table 2. The addition of unfermented or *Ganoderma*‐fermented corncobs did not significantly change the contents of protein (range: 76–79 g kg^−1^ DW) and free sugar (2 g kg^−1^ DW), though it minimally affected lipid content (range 123–144 g kg^−1^ DW in breadsticks with B73 and RR corncobs fermented or not with *Ganoderma versus* 145 g kg^−1^ DW of STD). Differently, the content of ash increased when breadsticks were enriched with fermented corncobs (B73 + G, 33 g kg^−1^ DW; RR + G, 32 g kg^−1^ DW *versus* 31 g kg^−1^ DW of the other breadsticks). Furthermore, corncob addition significantly increased total dietary fiber (by about nine times *versus* STD) and decreased starch contents (by about 11% *versus* STD). The characterization of the dietary fiber fractions showed consistent enrichment of IDF content due to the presence of corncobs (B73, RR), but also significant increase in SDF contents in breadsticks containing the *Ganoderma*‐fermented corncobs (B73 + G, RR + G), with respect to the unfermented samples (Table 2). No significant differences were found in RS contents for all breadstick samples. Based on the composition of the raw materials,^10^ the compositional changes of breadsticks were consistent with the replacement of 10 g of rice flour with the new ingredient.

Cirlincione *et al*.^39^ developed functional breads enriched with powdered *Pleurotus eringii*, analyzing the final products for vitamins and microelements. Their findings revealed an increase in vitamins B1, B2, B3 and D as well as in calcium, phosphorus, selenium and potassium. In this context, considering the mineral and vitamin composition of *Ganoderma*, further research will be conducted to determine the micronutrient content of breadsticks, aiming to highlight other interesting functional properties.

### Starch digestibility

**Figure 4:**
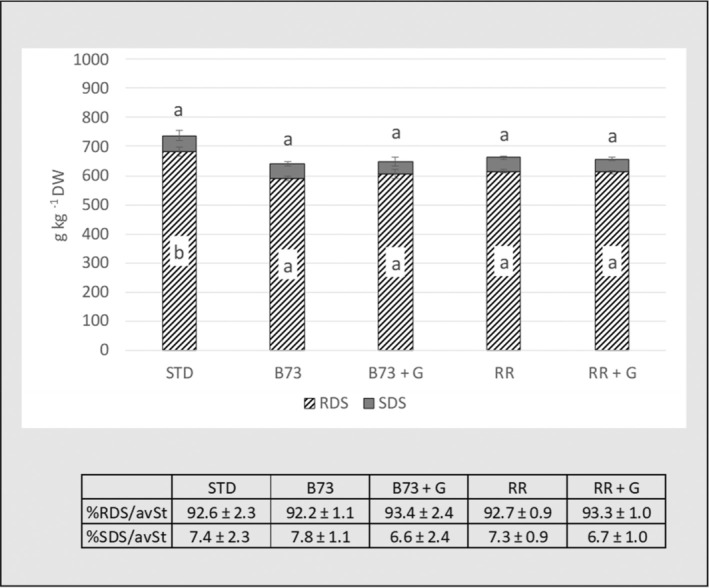
Contents of RDS and SDS (g kg^−1^ DW) and percentage of RDS and SDS on total available starch, in breadsticks samples. STD, breadsticks not containing corncobs; B73, breadsticks with B73 corncobs; B73 + G, breadsticks added with *Ganoderma‐*fermented B73 corncobs; RR, breadsticks with RR corncobs; RR + G, breadsticks added with *Ganoderma* fermented RR corncobs. Data reported as mean ± SD (*n* = 3). RDS, rapidly digestible starch; SDS, slowly digestible starch; %RDS/avST, percentage of RDS on total available starch; %SDS/avST, percentage of SDS on total available starch. Within the same variable, data not sharing common letters are significantly different (*P* < 0.05).

Results of starch digestibility of breadsticks are shown in Fig. 4, where data are expressed as grams of RDS and SDS per kilogram DW of samples and as percentage of RDS and SDS on total available starch (avSt). The presence of unfermented or *Ganoderma*‐fermented corncobs did not change the rate of digestibility of the available starch: %RDS/avSt and %SDS/avSt of breadsticks were unaffected by the addition of the new ingredients, suggesting that the enrichment in dietary fiber did not alter the accessibility of rice starch to amylolytic enzymes.

Maize cobs are a lignocellulose biomass, characterized by a close intertwining of cellulose, hemicellulose and lignin, whose total content represents up to 94% of the corncob on a dry basis.^40^, ^41^ In fact, the enrichments of dietary fiber found in breadsticks formulated with the new ingredients are ascribed mainly to the insoluble fraction and only to a little percentage of soluble fraction due to *Ganoderma* growth. The addition of sources of dietary fiber (either SDF or IDF) z to starchy food has been explored in the literature to limit the postprandial glycemic impact of food consumption: in this context, an inhibitory action on *in vitro* starch digestibility was found in the case of IDF.^42^, ^43^ As regards SDF, other proposed mechanisms involve the increase in viscosity that influences gastric emptying, nutrient diffusion, interaction between starch and enzymes, the competition with water holding that limits starch gelatinization and starch granule coating properties.^44^, ^45^ The mechanisms hypothesized are related to an inhibitory effect on *α*‐amylase, a steric hindrance that limits accessibility to enzyme into starch granule and an interference action on starch granule gelatinization.^46^ The effect of IDF in food seems less consistent: in bread formulated with refined wheat flour the addition of 300 and 450 g kg^−1^ of whole grain buckwheat flour containing 217 g kg^−1^ of TDF decreased the hydrolysis index of starch by 9% and 17% with respect to control white bread, possibly due to the inhibitory effect of flavonoid on amylolytic enzymes.^47^ Similarly, bread prepared with white flour with added ginseng IDF (0–80 g kg^−1^ flour) resulted in a dose‐dependent decrease in glucose release after *in vitro* starch digestion in comparison with control, possibly due to the high total phenolic content of ginseng extract (8.68 ± 0.20 g kg^−1^).^48^ Differently, the replacement of 30% lipid by IDF in sponge cake did not affect starch digestibility,^49^ as well as the addition of bran (100–300 g kg^−1^) to pasta prepared from durum wheat semolina.^50^ A further study was conducted by Liu *et al*.^51^ which analyzed the starch digestibility in rice noodles formulated by blending rice flour with different concentration of IDF extracted from rice bran (0–120 g kg^−1^). Their results showed that a 30 g kg^−1^ bran enrichment significantly decreased RDS and SDS fractions while increasing RS ones compared to control. However, a 120 g kg^−1^ bran enrichment resulted in higher RDS and SDS and lower RS with respect to control. The authors ascribed this latter effect to the disruption of starch structure of noodles. In the same study, the effects of single IDF polymers, such as hemicellulose, lignin and cellulose, were investigated displaying for cellulose the lowest ability of increasing SDS and RS and the weakest barrier effect on glucose diffusion. Overall, these studies underline that the digestibility of starch is influenced by multiple factors including the type and the quantity of dietary fiber and the structure of the food matrix. In the breadsticks enriched with 100 g kg^−1^ corncobs developed in the present study, dietary fiber was insoluble and predominantly cellulosic, with less than 10 g kg^−1^ SDF for samples containing the *Ganoderma*‐fermented corncobs; the ingredient had a hard consistency (as confirmed by the texture of the experimental breadsticks; see Table 1) and the structural features were similar among samples, so it is plausible that the dietary fiber–starch interaction was limited as was the interference on glucose release, and this could justify the absence of any dietary fiber effects on starch digestibility in our samples.

However, it is noteworthy that the absolute quantities of RDS of the experimental samples decreased in the breadsticks added with unfermented or fermented corncobs, because of their lower total starch content compared to the STD breadsticks (Table 2 and Fig. 4). This allows us to hypothesize that the consumption of an equivalent portion of breadsticks formulated with the new ingredient could have a lower postprandial glycemic impact than the STD one.

### Phenol content and antioxidant activity

**Figure 5:**
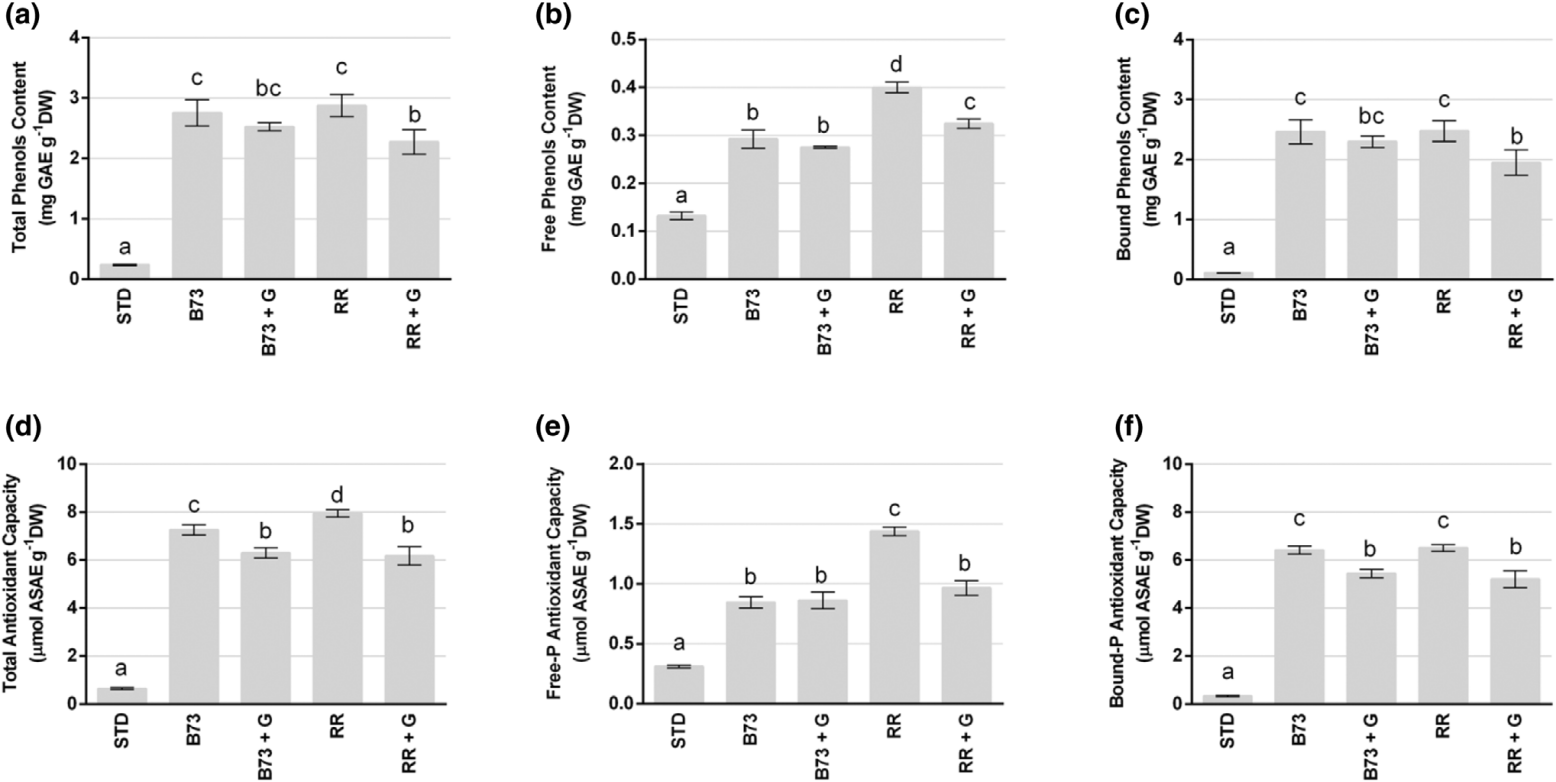
Total phenolic content (mg GAE g^−1^ DW) and antioxidant capacity (nmol ASAE g^−1^ DW) in breadsticks. Content of (a) total, (b) free and (c) bound phenolics, and antioxidant capacity in (d) total, (e) free and (f) bound phenol fractions from different breadsticks: without corncobs (STD), enriched with B73 or with RR corncobs, enriched with *Ganoderma*‐fermented B73 (B73 + G) or RR (RR + G) corncobs. Values represent the mean ± SD (*n* = 3). Different letters indicate statistically significant differences (*P* < 0.05) assessed with two‐way ANOVA.

Figure 5 illustrates the content of the total, free and bound phenolic fractions (Fig. 5(a)–(c)) and the relative antioxidant capacity (Fig. 5(d)–(e)) in gluten‐free breadsticks containing the B73 or RR corncobs or the corresponding *Ganoderma*‐fermented corncobs, compared to STD. The total phenolic content and antioxidant capacity of STD breadsticks were found to be very low (0.24 mg GAE g^−1^ DW and 0.64 μmol ASAE g^−1^ DW, respectively), with no discernible difference in levels and activity between free and bound phenolics. The incorporation of unfermented or *Ganoderma*‐fermented corncobs into the dough resulted in a notable enhancement in the total phenol content and antioxidant capacity of the resulting breadsticks. Consistently with what was previously reported for the composition of maize and fermented cobs,^10^ in breadsticks containing these ingredients the main phenolics were represented by the bound ones, whose levels were much higher (about 2.5 mg GAE g^−1^ DW for B73 and RR, 2.25 mg GAE g^−1^ DW for B73 + G and 1.95 mg GAE g^−1^ DW for RR + G) than those of the free phenolics (about 0.3 mg GAE g^−1^ DW for B73 and B73 + G, 0.40 mg GAE g^−1^ DW for RR and 0.32 mg GAE g^−1^ DW for RR + G). The same behavior was observed for the antioxidant capacity, with higher values associated with the bound phenolic fraction (about 6.5 μmol ASAE g^−1^ DW for B73 and RR, 5.44 μmol ASAE g^−1^ DW for B73 + G and 5.21 μmol ASAE g^−1^ DW for RR + G) than with the free ones (about 0.8 μmol ASAE g^−1^ DW for B73 and B73 + G, 1.44 μmol ASAE g^−1^ DW for RR and 0.97 μmol ASAE g^−1^ DW for RR + G). The breadsticks containing the *Ganoderma*‐fermented corncobs exhibited significantly lower levels of phenols and antioxidant capacity when compared to the breadsticks containing only corncobs. The results were more pronounced in the case of RR + G breadsticks than in the case of B73 + G breadsticks. This finding is consistent with a previous study carried out on the ingredients,^10^ which indicated that *Ganoderma* growth was accompanied by a decrease of phenolic compounds and that it occurred more rapidly on RR than on B73 corncobs. Finally, the breadsticks containing RR corncobs exhibited the highest content of free phenolics and highest total antioxidant capacity.

In recent years, there has been a notable focus on the sustainability of agricultural practices and the food system as a whole. This has involved exploring the potential of waste byproducts as a source of non‐nutritive bioactive compounds that can be used as health‐promoting food ingredients.^52^, ^53^ Agricultural waste byproducts provide food with fibers, which are important for gut health, and phenols, which are important for their antioxidant and free radical scavenging properties.^54^ In food, some of the phenolic compounds are present in a bioavailable (extractable) form that can be dissolved in the stomach and absorbed by the small intestine, while most of the polyphenols are associated with dietary fiber, forming complex conjugates in the food matrix and remaining intact down to the lower gastrointestinal tract. These conjugates, which have been defined as ‘antioxidant dietary fiber’, are of interest due to their capability to exert systemic effects. The intestinal microbiota can catabolyze the antioxidant dietary fiber resulting in a gradual release of the bioactive compounds. These metabolites can be partially absorbed or remain in the gut tissues where they act as antioxidants and promote gut homeostasis.^55^, ^56^

Our results suggest that, in general, the addition of unfermented or *Ganoderma*‐fermented maize cobs to dough results in increased levels of phenolic compounds with antioxidant properties in the breadsticks, thus adding nutritional value to the final gluten‐free product. The characteristics of the various corncob‐based food matrices analyzed were consistent with those of the unfermented or *Ganoderma*‐fermented corncob ingredients, as reported by Castorina *et al*.^10^ This indicated a significant abundance in the food matrix of the bound phenolic fraction, which includes not only the phenolics covalently bound to cell wall components, but possibly the non‐extractable ones, namely those forming arrays with the dietary fibers of the matrix, also through non‐covalent interactions, or those trapped by them, a phenomenon that may occur as a consequence of food processing.^55^ In particular, the RR and RR + G breadsticks, in addition to showing high contents of bound (non‐extractable) phenols, also exhibited the highest content of free (extractable) phenols, which may be associated with the low abundance of TDF and of IDF in these food matrices (see Table 2). Moreover, RR cobs showed a characteristic red pigmentation due to the presence in the glumes of flavonoids (anthocyanins and/or phlobaphenes^10^). A recent study conducted on mice indicated that the effect of the intake of anthocyanin‐ and phlobaphene‐enriched maize diets suggests a role of flavonoids in modulating the gut microbiota and in alleviating dextran sodium sulfate‐induced colitis.^56^

In this view, RR corncobs appear to be the most promising ingredient for the formulation of functional food providing various active compounds whose accessibility/bioavailability might be differentially modulated by the matrices with a positive impact along the entire gastrointestinal pathway. This aspect, together with a more detailed analysis of the phenolic composition of the cobs and the effect of fermentation on the specific compounds, is of paramount interest and deserves further investigation.

Moreover, the gluten‐free food products that are created to satisfy the nutritional needs of consumers suffering from celiac disease, a chronic autoimmune reaction to gluten, tend to have poor nutritional values in terms of micronutrients, protein content and fiber.^57^, ^58^ The use of maize cobs, either fermented with *Ganoderma annularis* or not, in the formulation of gluten‐free products, as well as contributing to the practice of recycling agricultural waste within a circular economy framework, may represent an innovative strategy for enriching foods with functional metabolites, thereby meeting needs in a public health context.

## CONCLUSION

This study investigates the potential of using corncobs either fermented with *Ganoderma annularis* or not to enhance the nutritional value of gluten‐free breadsticks. Corncobs used were from the maize inbred line B73 and RR, a locally cultivated landrace from northern Italy. SSFs were carried out with *Ganoderma* on either B73 or RR corncobs, and the entire cultures, dried and ground, were used as a functional ingredient in gluten‐free breadstick production.

Corncob addition significantly increased TDF (by about nine times *versus* STD) and decreased starch contents (by about 11% *versus* STD). Moreover, corncobs and particularly the fermented ones, increased the TDF and SDF content. The addition of unfermented or *Ganoderma*‐fermented corncobs to breadstick formulations also resulted in a notable enhancement of the total phenol content. As regards the type of corncobs used, the RR landrace appears the most interesting ingredient, providing the highest levels of free phenols and antioxidant capacity. In this view, RR corncobs appear to be the most promising ingredient for the formulation of functional food providing various active compounds whose accessibility/bioavailability might be differentially modulated by the matrices with a positive impact along the entire gastrointestinal pathway. This interesting aspect will be the object of further investigation.

During the scale‐up to an industrial pilot plan production thanks to the larger quantity of sample available, the effect of adding the new ingredient on dough workability should be considered, including the rheological properties of the dough, such as extensibility, elasticity and stickiness. Further studies should also investigate the possibility of increasing the amount of the functional ingredient to be added in breadstick formulation at concentration higher than 100 g kg^−1^ to fully exploit the nutritional features of the new ingredient. Moreover, consumer acceptability towards the developed food products should be also taken into consideration.

Overall, *Ganoderma*‐fermented corncobs may enhance the fiber and antioxidant content of gluten‐free breadsticks, suggesting improved health benefits. The proposed approach was effective in converting a byproduct (corncob) into a matrix that was used as a functional ingredient to increase the nutritional profile of gluten‐free breadsticks, without compromising their technological features, thus contributing to widening the range of baked goods consumable by gluten‐intolerant people.

## CONFLICT OF INTEREST

The authors declare no conflicts of interest.

## Data Availability

Data are available on request from the authors.
